# Venous Thromboembolism among Hospitalized Patients with COVID-19 Undergoing Thromboprophylaxis: A Systematic Review and Meta-Analysis

**DOI:** 10.3390/jcm9082489

**Published:** 2020-08-03

**Authors:** Gerald Chi, Jane J. Lee, Adeel Jamil, Vamsikrishna Gunnam, Homa Najafi, Sahar Memar Montazerin, Fahimehalsadat Shojaei, Jolanta Marszalek

**Affiliations:** 1 Division of Cardiovascular Medicine, Department of Medicine, Beth Israel Deaconess Medical Center, Harvard Medical School, Boston, MA 02215, USA; vgunnam@bidmc.harvard.edu (V.G.); drnajafihoma@gmail.com (H.N.); sahar632nahal@gmail.com (S.M.M.); fahimeh.sadat.shojaei@gmail.com (F.S.); 2 Baim Institute for Clinical Research, Boston, MA 02215, USA; jane.lee@baiminstitute.org; 3 Department of Medicine, OSF Saint Francis Medical Center, Peoria, IL 61637, USA; md.adeeljamil@gmail.com; 4 Department of Neurology, David Geffen School of Medicine at UCLA, Ronald Reagan UCLA Medical Center, Los Angeles, CA 90095, USA; jmarszalek.md@gmail.com

**Keywords:** venous thromboembolism, pulmonary embolism, deep vein thrombosis, thromboprophylaxis, COVID-19

## Abstract

Background: Preliminary evidence indicates that prophylactic-dose thromboprophylaxis may be inadequate to control the increased risk of venous thromboembolism (VTE) in patients hospitalized for coronavirus disease 2019 (COVID-19) infection. Additionally, it remains unclear whether the D-dimer measurement is useful for VTE risk stratification among COVID-19 patients. This study aimed to offer benchmark data on the incidence of VTE and to examine the difference in D-dimer levels among anticoagulated COVID-19 patients with and without VTE incident. Methods: A comprehensive literature review of PubMed from inception to May 2020 was performed for original studies that reported the frequency of VTE and death among COVID-19 patients who received thromboprophylaxis on hospitalization. The endpoints included VTE (a composite of pulmonary embolism (PE) or deep vein thrombosis (DVT)), PE, DVT, and mortality. Results: A total of 11 cohort studies were included. Among hospitalized COVID-19 patients, 23.9% (95% confidence interval (CI), 16.2% to 33.7%; I^2^ = 93%) developed VTE despite anticoagulation. PE and DVT were detected in 11.6% (95% CI, 7.5% to 17.5%; I^2^ = 92%) and 11.9% (95% CI, 6.3% to 21.3%; I^2^ = 93%) of patients, respectively. Patients in the intensive care unit (ICU) had a higher risk for VTE (30.4% )95% CI, 19.6% to 43.9%)) than those in the ward (13.0% (95% CI, 5.9% to 26.3%)). The mortality was estimated at 21.3% (95% CI, 17.0% to 26.4%; I^2^ = 53%). COVID-19 patients who developed VTE had higher D-dimer levels than those who did not develop VTE (mean difference, 2.05 µg/mL; 95% CI, 0.30 to 3.80 µg/mL; *P* = 0.02). Conclusions: The heightened and heterogeneous risk of VTE in COVID-19 despite prophylactic anticoagulation calls into research on the pathogenesis of thromboembolic complications and strategy of thromboprophylaxis and risk stratification. Prominent elevation of D-dimer may be associated with VTE development and can be used to identify high-risk subsets.

**PROSPERO Registration Number:** CRD42020189192

## 1. Introduction

Patients with coronavirus disease 2019 (COVID-19) infection may present with a diverse clinical spectrum, ranging from asymptomatic carriage, mild respiratory or gastrointestinal symptoms, to severe pneumonia and multi-organ failure [1,2]. In some cases, COVID-19 infection may be associated with abnormalities in coagulation parameters (e.g., D-dimer, prothrombin time, partial thromboplastin time, fibrin/fibrinogen degradation products, and platelet counts) that are consistent with infection-induced inflammatory changes as observed in disseminated intravascular coagulopathy [3]. COVID-19-associated coagulopathy has been associated with adverse prognosis, including admission to intensive care unit (ICU), the need for ventilatory support, and mortality [4,5,6,7]. Interim guidance from the International Society of Thrombosis and Hemostasis suggests monitoring D-dimer, fibrinogen, prothrombin time, and platelet count to determine prognosis and assist management decision in COVID-19 patients requiring hospitalization [8]. Deterioration of coagulation parameters may warrant more aggressive critical care support and consideration for experimental therapies and blood product support. Conversely, stepdown of treatment can be considered if the clinical condition and coagulation markers have stabilized or improved.

Pharmacological thromboprophylaxis has shown promise in preventing venous thromboembolism (VTE) for high-risk individuals. In acutely ill hospitalized patients, the incidence of VTE ranges from 5% to 15%, and can be effectively reduced by one-half to two-thirds with appropriate thromboprophylaxis [9]. In critically ill patients, the incidence of deep vein thrombosis (DVT) ranges from 13% to 31% without thromboprophylaxis [10]. Similarly, the risk may be reduced to 10.9% to 15.5% with low molecular weight heparin (LMWH) or low-dose unfractionated heparin (UFH) [11,12,13,14]. Patients with coronavirus disease 2019 (COVID-19) pneumonia may be predisposed to thrombotic complications due to excess inflammatory response, endothelial dysfunction, platelet activation, and stasis of blood flow [15]. In view of the increased risk of VTE among patients hospitalized for COVID-19 pneumonia, current guidance statements recommend the use of standard-dose thromboprophylaxis with UFH or LMWH [16,17,18]. However, the adequacy of standard thromboprophylaxis is challenged by emerging studies reporting a high incidence of VTE among anticoagulated patients. A report from France revealed that compared to the matched historic non-COVID-19 cohort, COVID-19 patients with acute respiratory distress syndrome had a five-fold risk of pulmonary embolism (PE) despite anticoagulation (11.7% vs. 2.1%), among which 2.7% developed hemorrhagic complications [19]. Another study from the Netherlands demonstrated that despite routine standard thromboprophylaxis, 20% of COVID-19 patients developed VTE (47% in the ICU and 3.3% in the ward setting) at seven days [20].

The true incidence of VTE and the optimal thromboprophylaxis strategy for hospitalized COVID-19 patients remain unsettled. To address the evidence gap, the study aimed to synthesize the current evidence on the incidence of VTE among COVID-19 patients undergoing thromboprophylaxis in the ICU or ward setting, and to examine the potential prognostic value of the D-dimer measurement.

## 2. Methods

This study was conducted in accordance with the Preferred Reporting Items for Systematic Reviews and Meta-Analyses (PRISMA) guidelines, and the protocol was registered in PROSPERO (registration number: CRD42020189192).

### 2.1. Search Strategies and Selection Criteria

A systematic literature search was performed in PubMed, supplemented by a hand search of references from relevant publications. The search terms were organized in thematic building blocks that could be combined as required. A detailed search strategy is provided in Table S1. Human studies, published as original research articles, letters, or brief reports that reported the incidence of study endpoints (VTE, PE, deep vein thrombosis (DVT), or mortality) among hospitalized patients with laboratory-confirmed COVID-19 infection who received at least standard doses of thromboprophylaxis with UFH or LMWH were included. All searches were limited to the English language and the time from inception to 31 May 2020.

### 2.2. Data Extraction

Data extracted from each study included study design, population, clinical setting (ICU or ward), thromboprophylaxis strategy, patient characteristics, D-dimer levels, follow-up duration, and frequencies for VTE events or deaths. Database search, article screening, and study selection were performed independently by two investigators using a standardized approach. Disagreement in extracted data was adjudicated by a third investigator. A flow diagram depicting the process of the literature search and screening is provided in Figure S1.

### 2.3. Quality Assessment

Two independent investigators assessed the quality of cohort studies in accordance with the Newcastle–Ottawa Scale. Disagreement in the quality assessment was resolved by discussion and consensus. The quality assessment criteria and forms were provided in Tables S2 and S3.

### 2.4. Study Endpoints

Study endpoints include: (1) VTE, defined as a composite of PE or DVT; (2) PE as detected by computed tomography pulmonary angiography in the case of clinical suspicion and/or acute degradation of hemodynamic or respiratory status; (3) DVT of the lower or upper extremity, as detected by ultrasonography in the case of clinical suspicion or from screening; and (4) all-cause mortality.

### 2.5. Statistical Analysis

The first part of the analysis aimed to estimate the incidence of VTE and mortality among hospitalized COVID-19 patients who received thromboprophylaxis. The proportion was deemed as the effect size, and calculated as the number of cases (VTE, PE, DVT, and mortality) divided by the size of the sample. Considering the distribution of observed proportions was skewed and not centered around 0.5, logit transformation was performed to conform to the normal distribution [21]. For each study, the natural logarithm of the proportion was used as an effect size statistic, and the inverse variance of the transformed proportion was used as study weight. Confidence intervals (CIs) of proportions were calculated by normal approximation. The summary effect size was then computed by fitting a random-effects model using the DerSimonian and Laird method. Heterogeneity across the studies was assessed using the Q test (with the threshold of *P* < 0.10 indicating the presence of heterogeneity) and I^2^ statistic (with the values of 0.25, 0.50, and 0.75 indicating a low, moderate, and high degree of heterogeneity, respectively). A subgroup analysis by clinical setting was performed to compare the observed effect size in the ICU setting vs. the ward setting and to test its potential influence on heterogeneity. Funnel plots and an Egger’s test were employed to detect small-study effects.

The second part of the analysis aimed to compare the D-dimer level between cases and controls. Two endpoints were studied: VTE and mortality. Cases were defined as patients who developed VTE or died, whereas controls were defined as patients who did not develop VTE or survived. The absolute difference in the mean D-dimer value was deemed as the effect size. For each study, the D-dimer was uniformly expressed as µg/mL, and the mean and standard deviation (SD) were estimated from the median, interquartile range, and sample size [22]. The overall effect size (i.e., mean difference of D-dimer level) was summarized using the inverse variance weighted approach in a random-effects model, with a positive value indicating a higher average level of D-dimer in cases than in controls.

Analysis was performed using the meta package in the R software (Version 3.5.2; the R Foundation for Statistical Computing), and Review Manager (Version 5.3; the Nordic Cochrane Centre, the Cochrane Collaboration).

## 3. Results

A total of 1981 subjects from 11 studies were included in the meta-analysis and summarized in Table 1 [19,20,23,24,25,26,27,28,29,30,31]. The majority of studies were retrospective, except for two studies. Five studies were conducted exclusively in the ICU setting, whereas six studies enrolled patients in the ward setting, with 10% to 38% requiring admission to the ICU. The mean age was consistently between 60 and 70 years. The proportion of males ranged from 52% to 81%. Approximately 1% to 7% patients had a history of VTE, and up to 10% of patients had cancer. The follow-up duration varied from 6 to 55 days. Only one study reported bleeding outcomes, in which 4 out of 150 patients (2.7%) had hemorrhagic complications [19]. The quality of the studies was generally high, with scores ranging from 6 to 9 as evaluated with the Newcastle–Ottawa Scale (Tables S2 and S3).

### 3.1. Incidence of VTE

**Figure 1:**
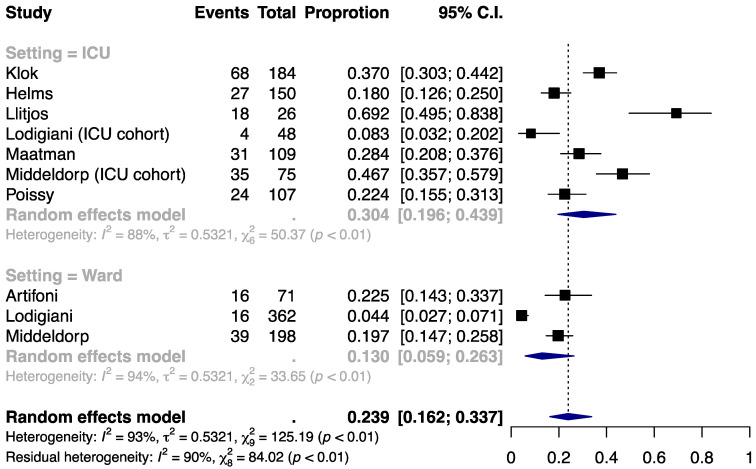
Incidence of venous thromboembolism (VTE) by clinical setting.

The proportion of COVID-19 patients who underwent thromboprophylaxis and developed VTE was estimated at 23.9% (95% CI, 16.2% to 33.7%; Figure 1). The incidence was higher in the ICU setting (30.4% (19.6% to 43.9%)) than in the ward setting (13.0% (5.9% to 26.3%)). Substantial heterogeneity was present within and across the subgroups (I^2^ = 88%, 94%, and 93% for ICU, ward, and overall) and could not be explained by the clinical setting (residual I^2^ = 90%). No significant small-study effect was noted by the funnel plot (Figure S2) or Egger’s test (*P* = 0.89; Table S4).

### 3.2. Incidence of PE

**Figure 2:**
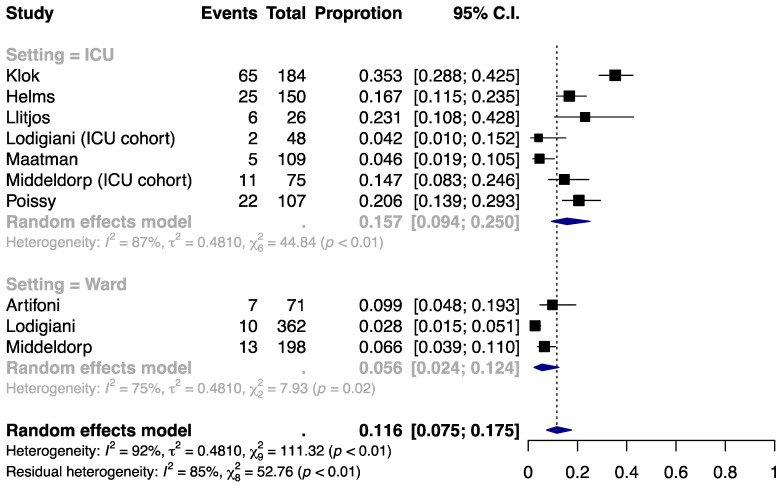
Incidence of pulmonary embolism (PE) by clinical setting.

Approximately 11.6% (95% CI, 7.5% to 17.5%) of the anticoagulated COVID-19 patients developed PE (Figure 2). Similar to VTE, the incidence was higher in the ICU setting (15.7% (9.4% to 25.0%)) than in the ward setting (5.6% (2.4% to 12.4%)). A high degree of heterogeneity was present within and across the subgroups (I^2^ = 87%, 75%, and 92% for ICU, ward, and overall) and could not be explained by the clinical setting (residual I^2^ = 85%). No significant small-study effect was noted by the funnel plot (Figure S3) or Egger’s test (*P* = 0.06; Table S4).

### 3.3. Incidence of DVT

**Figure 3:**
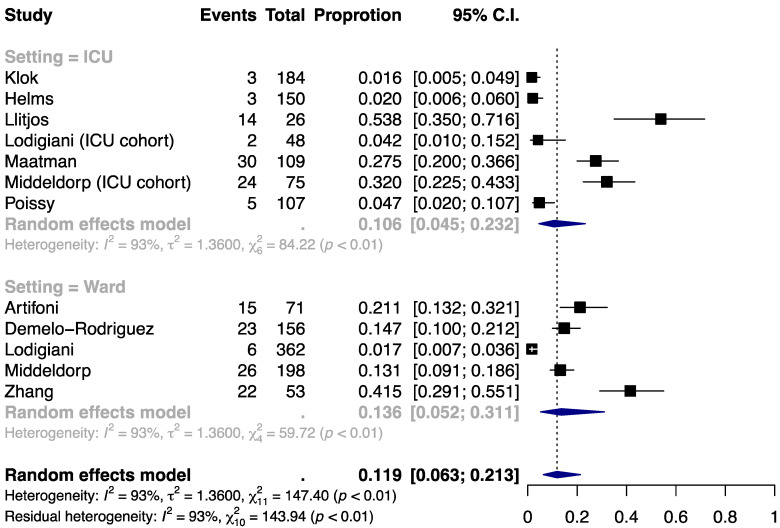
Incidence of deep vein thrombosis (DVT) by clinical setting.

The incidence of DVT was estimated at 11.9% (95% CI, 6.3% to 21.3%) among COVID-19 patients with thromboprophylaxis (Figure 3). The proportion was similar between the ICU subgroup (10.6% (4.5% to 23.2%)) and the ward subgroup (13.6% (5.2% to 31.1%)). Similarly, substantial heterogeneity was present within and across the subgroups (I^2^ = 93%, 93%, and 93% for ICU, ward, and overall) and could not be explained by the clinical setting (residual I^2^ = 93%). Funnel plot asymmetry was noted (Figure S4) and the Egger’s test suggested small-study effects (*P* = 0.0012; Table S4).

### 3.4. Mortality

Six studies were available for analysis of all-cause mortality (Figure S5). Mortality ranged from 11.5% to 30.3%, and was estimated at 21.3% (95% CI, 17.0% to 26.4%) among COVID-19 patients with thromboprophylaxis. There was a moderate heterogeneity across the studies (I^2^ = 53%). No significant small-study effect was noted by the funnel plot (Figure S6) or Egger’s test (*P* = 0.18, Table S4).

### 3.5. Difference in D-dimer between Cases and Controls

Mean D-dimer levels between cases and controls were compared (Figure S7). Compared with patients who did not develop VTE, a higher level of D-dimer was observed in patients who developed VTE (mean difference, 2.05 µg/mL; 95% CI, 0.30 to 3.80 µg/mL; *P* = 0.02). The mean difference was not significant between non-survivors and survivors (mean difference, 3.70; 95% CI, −2.32 to 9.72; *P* = 0.23). Taken together, patients who developed VTE or died had higher D-dimer levels than those who did not develop VTE or survived (mean difference, 2.57; 95% CI, 1.01 to 4.14; *P* = 0.001). The I^2^ values were 64% for VTE and 96% for mortality, suggesting a moderate-to-high and high degree of heterogeneity.

## 4. Discussion

Based on aggregate data from cohort studies, the meta-analysis provides benchmark information on the incidence of VTE among COVID-19 patients who received thromboprophylaxis (summarized in Figure S8). Overall, approximately 24% developed VTE (12% developed PE and 12% developed DVT) despite anticoagulation with at least prophylactic dosing. The risks of VTE and PE were both higher in the ICU setting (30% and 16%) than in the ward setting (13% and 6%). In contrast, the risk of DVT was similar in the two settings. The heightened risk of VTE points to an unmet need for intensified or extended thromboprophylaxis for high-risk hospitalized COVID-19 patients, which corroborates with the recent advocacy of intermediate-dose LMWH (e.g., enoxaparin, 40–60 mg daily or 40 mg twice daily, especially for BMI > 30 kg/m^2^) by institutional antithrombotic protocols [32]. Only 1 of the 11 included studies reported bleeding outcomes. Unlike the acutely ill medical patients [33], the benefit-risk tradeoff (i.e., preventing VTE while maintaining an acceptable risk of bleeding) of anticoagulation has not been formally studied in the context of hospitalized COVID-19 patients. Several randomized trials are underway to investigate the efficacy and safety of intermediate- or therapeutic-dose versus prophylactic-dose LMWH in hospitalized COVID-19 patients, such as COVID-HEP (ClinicalTrials.gov Identifier: NCT04345848), IMPROVE (ClinicalTrials.gov Identifier: NCT04367831), and HEP-COVID (ClinicalTrials.gov Identifier: NCT04401293). Results from these studies will inform antithrombotic strategies for COVID-19 patients.

There are important caveats to heed when interpreting the data on VTE incidence among COVID-19 patients. Pulmonary vessel occlusion in COVID-19 patients might result from embolism, thrombosis, or the combination of both. On pulmonary angiography, filling defects of pulmonary vessels may represent reminiscent of in situ microthrombi that also exist in non-pulmonary organs and constitute a part of multi-organ failure. [34] As the occlusion detected by CTPA may not be exclusively caused by embolism, the incidence of PE from the present analysis could be overestimated. Autopsy studies have reported pulmonary vessel occlusion of embolic and thrombotic origin in COVID-19 patients. Specifically, Wichmann et al. described massive PE associated with fresh thrombi in the prostatic venous plexus among one-third of autopsied patients [35]. In contrast, Lax et al. described thrombotic occlusion of small and mid-sized pulmonary arteries without apparent embolism in all autopsied patients [36]. Unlike macro-thrombosis in PE that is featured by excessive thrombin generation and aggravated by the imbalance between pro- and anti-coagulant factors, pulmonary micro-thrombosis is hallmarked by altered alveoli and pulmonary microvasculature associated with platelet/ultra-large von Willebrand factor multimers anchored to the injured endothelium and intra-alveolar fibrin deposition, which develops as a consequence of endotheliopathy due to direct viral infection and immune-mediated host response [37,38]. Therapeutic implications of distinctive pathogenesis between pulmonary embolism and pulmonary thrombosis should be further investigated. As anticoagulation is generally not indicated for the treatment of thrombotic microangiopathy with the exception of antiphospholipid syndrome [39], aggressive thromboprophylaxis may not translate into improved efficacy but pose an increased risk of hemorrhagic complications [34].

Substantial heterogeneity was observed in the VTE composite and its components. The heterogeneity was not mitigated by stratification by clinical setting (residual I^2^ > 75%), and may be related to the different VTE risk profile and clinical severity of patients across the studies. The heterogeneity in VTE risk suggests that the COVID-19 patients may represent a population with heterogeneous predisposition to thrombosis. Alternatively, it may reflect the difference in the methodology employed to identify VTE across the studies on COVID-19 patients. For instance, the indications for VTE detection could differ among the studies, and include clinical parameters (e.g., worsening PaO2/FiO2 despite inhaled nitric oxide or after prone positioning, hemodynamic deterioration requiring fluid challenge and/or increased norepinephrine infusion rate, or dilated right ventricle without acute cor pulmonale), laboratory assessment (e.g., significant or rapid elevation of D-dimer values despite anticoagulation), or both (Table 1). Furthermore, the timing and frequency of performing diagnostic imaging could also contribute to heterogeneity. This observation highlights the need for standardized VTE diagnostic and management approaches to hospitalized or critically ill COVID-19 patients.

Thus far, few studies provided insights into baseline VTE risk assessment of hospitalized COVID-19 patients. A report of a nationwide dataset from China showed that 40% of COVID-19 patients were at high risk for VTE as evaluated by the Padua prediction score (PPS) [40]. Another single-institution study from China reported that 66% of the COVID-19 patients were in the high-risk group (PPS ≥ 4), and DVT was identified more frequently in the high-risk group than the low-risk patients (62.8% vs. 14.3%) [31]. Furthermore, the combined use of CURB-65 score 3-5, PPS ≥ 4, and D-dimer > 1.0 µg/mL yielded a sensitivity of 88.5% and specificity of 61.4% for DVT. Prior to the COVID-19 pandemic, the incorporation of D-dimer into VTE risk assessment model has been shown to identify high-risk subsets that benefit from extended-duration thromboprophylaxis [41,42]. Although the D-dimer level is commonly elevated in COVID-19 infection [43], the present analysis demonstrated that COVID-19 patients who developed VTE had markedly higher D-dimer levels than those who did not develop VTE, with a mean difference of approximately 4 times the upper limit of normal (ULN). It should be noted that a conventional D-dimer cut-off of 0.5 μg/mL may have inadequate specificity for predicting adverse outcomes or guiding anticoagulation decisions in COVID-19 patients. In a multicenter cohort study of adult inpatients with COVID-19, a D-dimer > 1.0 μg/mL was associated with 18-fold increased odds for in-hospital death, while the association was not significant with >0.5 μg/mL [5]. Additionally, the study by Tang et al. showed that the effect of heparin on reducing 28-day mortality was significant in patients with a D-dimer > 6-fold of ULN but not in the overall study population. However, D-dimer measurement may not be routinely performed in hospitalized patients. A recent meta-analysis indicated that other commonly available biomarkers (such as hemoglobin and albumin) could assist risk stratification for severe and fatal COVID-19 [44]. Decreased hemoglobin or albumin levels may reflect underlying inflammation or disease states that predispose patients to thrombosis. Moreover, D-dimer (>1.0 μg/mL), anemia (<12.5 g/dL for males; <11.0 g/dL for females), and hypoalbuminemia (<3.5 g/dL) were demonstrated to have comparable magnitude of association with VTE in acutely ill hospitalized patients who received thromboprophylaxis [45,46,47]. Further research is needed to investigate the incremental prognostic value of these biomarkers and the optimal cut-offs in COVID-19 patients.

### Limitations

Several limitations should be considered. First, information about the comparative efficacy and safety of thromboprophylaxis strategies was unavailable. Second, most of the included studies were retrospective. Third, the decision to perform computed tomography pulmonary angiography or ultrasonography was at the discretion of the treating physicians, and ascertainment bias cannot be excluded. Fourth, the majority of the included studies evaluated the incidence of VTE events that occurred during hospital stay and followed the patients for a relatively short duration. However, a cohort study of medically ill hospitalized patients using administrative claims database showed that 56.6% of the VTE events occurred after discharge [48]. This finding is supported by another population-based study which demonstrated that approximately one-half of the VTE events were related to current or recent hospitalization, and a total of 75% of the VTE events developed after hospital discharge, with a median time to develop VTE of 19.5 days [49]. Indeed, the risk of VTE may extend beyond the standard course of anticoagulation, and VTE events may occur after hospital discharge due to lack of anticoagulation coverage and lead to recurrent hospitalization [50]. To better capture the time course of hospital-associated VTE, future studies should consider extending the follow-up duration to at least 90 days. Last, only two studies were available for examining the association of the D-dimer with mortality in the analysis. The insignificant difference in the D-dimer between non-survivors and survivors may reflect a lack of statistical power. Despite these limitations, the present meta-analysis represents a synthesis of the currently available data.

## 5. Conclusions

The heightened and heterogeneous risk of VTE in COVID-19 despite prophylactic anticoagulation calls for research on the pathogenesis of thromboembolic complications and the strategy of thromboprophylaxis and risk stratification. Marked elevation of D-dimer levels may be associated with VTE development and can be used to identify high-risk subsets. Prospective randomized clinical trials are warranted to investigate the comparative efficacy and safety of anticoagulation strategies.

## Figures and Tables

**Table 1:** Summary of included studies.

Author	Population	Setting	Thromboprophylaxis	Indication for VTE Detection	N	Age, year	Male, %	BMI, kg/m^2^	HTN, %	DM, %	Cancer, %	Smoking, %	History of VTE, %	ICU Admission, %	D-Dimer, μg/mL	Follow-up
Klok	COVID-19 patients admitted to the ICU	ICU	All patients received at least standard doses thromboprophylaxis (nadroparin 2850–5700 IU once daily to twice daily), with 9.2% receiving therapeutic anticoagulation at admission	CTPA/ultrasonography for PE/DVT if thrombotic complications were clinically suspected	184	64 ± 12	76	NR	NR	NR	2.7	NR	NR	100	NR	14 days
Helms	COVID-19 patients with acute respiratory distress syndrome admitted to the ICU	ICU	70% received prophylactic dosing (4000 UI/day for LMWH or if contraindicated, UFH at 5–8 U/kg/h) and 30% received therapeutic dosing	CTPA, either at the admission in ICU or during the stay, if PE was suspected based on clinical or laboratory parameters evolution	150	63 (53–71)	81.3	NR	NR	20	6	NR	5.3	100	2.27 (1.16–20)	7–30 days
Llitjos	Severe ICU COVID-19 patients	ICU	All patients were anticoagulated from admission: 31% (n = 8) with prophylactic anticoagulation and 69% (n = 18) with therapeutic anticoagulation	CTPA was performed systematically in patients with persistent hypoxemia or secondary deterioration. CDU was performed between day 1 and day 3 after admission. In patients without VTE during the first CDU, a second CDU was performed at day 7	26	68 (51.5–74.5)	77	NR	85	NR	0	27	4	100	1.750 (1.130–2.850)	NR
Maatman	Patients with laboratory-confirmed SARS-CoV-2 infection requiring intensive care	ICU	All patients admitted with COVID-19 receive VTE chemoprophylaxis including either 5000 U subcutaneous heparin every 8 h, 40 mg enoxaparin daily, or 30 mg enoxaparin bid	Extremity DVT was diagnosed on four-extremity duplex ultrasound performed for clinical suspicion for DVT. PE was diagnosed on contrast-enhanced cross-sectional imaging	109	61 ± 16	57	NR	68	39	NR	30	NR	100	0.506 (0.321–0.973)	36–55 days
Poissy	COVID-19 patients admitted to the ICU for pneumonia	ICU	All patients received thromboprophylaxis (UFH or LMWH)	CTPA was performed on suspicion of PE upon admission and/or acute degradation of hemodynamic or respiratory status	107	NR	NR	NR	NR	NR	NR	NR	NR	100	NR	6 days
Artifoni	Hospitalized patients with COVID-19	Ward	Daily administration of weight-appropriate enoxaparin following institutional recommendations (40 mg/day for BMI < 30 kg/m^2^, 60 mg/day for BMI 30 to 40 kg/m^2^ and 40 mg twice daily for BMI > 40 kg/m^2^) and covering the whole hospital stay	All patients were systematically examined for DVT by low limb venous duplex ultrasonography at hospital discharge or earlier if thrombosis was clinically suspected. Chest angio-CT scan was performed in case of suspicion of PE	71	64 (46.0–75)	60.6	27.3 (25.0–31.2)	41	20	6	9	7	18	0.79 (0.48–1.61)	13 days
Demelo-Rodríguez	Hospitalized in non-ICU with diagnosis of COVID-19 pneumonia and D-dimer > 1000 ng/ml	Ward	All but three patients received standard doses of thromboprophylaxis: enoxaparin 40 mg per day or bemiparin 3500 UI per day	Complete CUS was performed to screen for asymptomatic DVT	156	68.1 ± 14.5	65.4	26.9 ± 4.2	NR	NR	10.3	NR	1.3	10.3	2.148 (1.532–4.002)	9 days
Lodigiani	Adult symptomatic patients with laboratory-proven COVID-19	Ward/ICU	ICU cohort (n = 61): LMWH: The dosage was weight-adjusted in 17 patients and therapeutic in two patients on ambulatory treatment with direct oral anticoagulants General ward cohort (n = 327): 75% received initial in-hospital thromboprophylaxis: A prophylactic dosage was used in 41% of patients, 21% were treated with inter-mediate-dosage thromboprophylaxis, and 23% received therapeutic-dose anticoagulation	CTPA/ultrasonography were performed in subjects with signs or symptoms of DVT or with an unexplained clinical worsening of the respiratory function, primarily assessed using the PaO2/FIO2 ratio, or a rapid increase of D-dimer levels. Two-point CUS was used in the ICU; whole-leg ultrasound was performed in symptomatic patients in the general ward	388	66 (55–75)	68	NR	47.2	22.7	6.4	11.6	3.1	16	NR	18 days
Middeldorp	Hospitalized patients with COVID-19	Ward/ICU	Ward patients received prophylaxis with nadroparin 2850 IU once daily or 5700 IU for patients with a body weight of ≥100 kg. From April 3 onwards, patients in ICU received a double dose of nadroparin as compared to patients on the wards, which was nadroparin 2850 IU twice daily (bid) for patients with a body weight <100 kg and 5700 IU bid for those ≥100 kg	Based on concerns of a high risk of fatal VTE following early observations, during the follow-up period CUS screening was performed in the ICU every 5 days, while also performing a single cross-sectional round of CUS at the ward in the 10 days prior to data collection	198	61 ± 14	66	27 (24–31)	NR	NR	3.5	NR	5.6	38	1.1 (0.7–2.3)	7 days
Tang	Hospitalized patients with severe COVID-19.	Ward	Heparins (n = 99; 94 received 40–60 mg enoxaparin/d and 5 received UFH 10,000–15,000 U/d) vs. control (n = 350)	NR	449	65.1 ± 12.0	60	NR	NR	NR	NR	NR	NR	NR	1.94 (0.90–9.44)	28 days
Zhang	Critically ill adult patients with COVID-19	Ward	53 (37.1%) patients were given DVT prophylaxis with LMWH vs. control	CTPA was performed on suspicion of PE; ultrasound was performed to screen for DVT	143	63 ± 14	51.7	23.6 ± 3.0	39.2	18.2	4.9	6.3	0.7	10.5	2.7 (0.6–8.0)	24–55 days

**Abbreviations:** BMI, body mass index; CDU, complete duplex ultrasound; CUS, compression ultrasound; CTPA, computed tomography pulmonary angiogram; DM, diabetes mellitus; DVT, deep vein thrombosis; HTN, hypertension; ICU, intensive care unit; LMWH, low-molecular-weight heparin; NR, not reported; UFH, unfractionated heparin; VTE, venous thromboembolism.

## Supplementary Materials

The following are available online at https://www.mdpi.com/2077-0383/9/8/2489/s1, Figure S1: PRISMA flow diagram, Figure S2: Funnel plot of VTE, Figure S3: Funnel plot of PE, Figure S4: Funnel plot of DVT, Figure S5: Rate of mortality among COVID-19 patients undergoing thromboprophylaxis, Figure S6: Funnel plot of mortality, Figure S7: Mean difference in D-dimer between cases and controls, Figure S8: Summary of VTE incidence by clinical setting, Table S1: Search strategy, Table S2: Quality assessment criteria for cohort studies, Table S3: Quality assessment form for cohort studies, Table S4: Summary of Egger’s test for small-study effect.

## Abbreviations

**COVID-19**	coronavirus disease 2019
**DVT**	deep vein thrombosis
**LMWH**	low-molecular-weight heparin
**PE**	pulmonary embolism
**PPS**	Padua prediction score
**UFH**	unfractionated heparin
**VTE**	venous thromboembolism
