# Supplementary material for: Venous Thromboembolism among Hospitalized Patients with COVID-19 Undergoing Thromboprophylaxis: A Systematic Review and Meta-Analysis
Source: J Clin Med. 2020 Aug 3;9(8):2489. doi: 10.3390/jcm9082489 (PMC7463975; doi:10.3390/jcm9082489)

Supplementary Material

Venous Thromboembolism among Hospitalized Patients with COVID-19 Undergoing Thromboprophylaxis: A Systematic Review and Meta-Analysis

Gerald Chi, M.D.[1]; Jane J. Lee, Ph.D.[2]; Adeel Jamil, M.D.[3]; Vamsikrishna Gunnam, M.D.[1]; Homa Najafi, M.D.[1]; Sahar Memar Montazerin, M.D.[1]; Fahimehalsadat Shojaei, M.D.[1]; Jolanta Marszalek, M.D.[4]

[1] Division of Cardiovascular Medicine, Department of Medicine, Beth Israel Deaconess Medical Center, Harvard Medical School, Boston, MA, United States
[2] Baim Institute for Clinical Research, Boston, MA, United States
[3] Department of Medicine, OSF Saint Francis Medical Center, Peoria, IL, United States
[4] Department of Neurology, David Geffen School of Medicine at UCLA, University of California Los Angeles, Los Angeles, CA, United States

Table of Contents

Table S1. Search strategy
Table S2. Quality assessment criteria for cohort studies
Table S3. Quality assessment form for cohort studies
Table S4. Summary of Egger's test for small-study effect
Figure S1. PRISMA flow diagram
Figure S2. Funnel plot of VTE
Figure S3. Funnel plot of PE
Figure S4. Funnel plot of DVT
Figure S5. Rate of mortality among COVID-19 patients undergoing thromboprophylaxis
Figure S6. Funnel plot of mortality
Figure S7. Mean difference in D-dimer between cases and controls
Figure S8. Summary of VTE incidence by clinical setting

Supplementary Material

Table S1. Search strategy

**Query in PubMed**
(((coronavirus or corona-virus) AND (2019-nCoV or nCoV or COVID-19 or Covid19 or SARS-CoV* or SARSCov2 or ncov)) OR (pneumonia AND Wuhan) or "COVID-19" or "2019-nCoV" or "SARS-CoV" or SARSCOV2 or 2019-nCov or "2019 coronavirus" or "2019 corona virus" or covid19 or ncov OR "novel corona virus" or "new corona virus" or "2019 corona virus" OR "novel coronavirus" or "new coronavirus" or "2019 coronavirus”) AND (deep vein thrombosis OR pulmonary embolism OR venous thromb*) AND (thromboprophylaxis OR prophylaxis OR prevention OR anticoagula* OR heparin OR enoxaparin OR bemiparin OR nadroparin OR reviparin OR parnaparin OR certoparin OR dalteparin OR tinzaparin) AND English [lang]

Supplementary Material

Table S2. Quality assessment criteria for cohort studies

Domain	Item	Criterion	Rating
Selection	Representativeness of the Exposed Cohort (0 to 1)	Exposed individuals are representative in the community.	1
		No description.	0
	Selection of the Non-Exposed Cohort (0 to 1)	Drawn from the same community as the exposed cohort.	1
		No description.	0
	Ascertainment of Exposure (0 to 1)	Secure records (e.g. medical records/research records).	1
		No description.	0
	Demonstration That Outcome of Interest Was Not Present at Start of Study (0 to 1)	Yes	1
		No description.	0
Comparability	Matched or Adjusted by Key Factors (0 to 1)	Reported VTE risk factors.	1
	Matched or Adjusted by Additional Factors (0 to 1)	Reported D-dimer levels.	1
Outcome	Assessment of Outcome (0 to 1)	Independent or blind assessment, confirmation of the outcome by reference to secure records (e.g. medical records/research records), or record linkage (e.g. identified through ICD codes on database records).	1
		Self-reported records only, or not stated.	0
	Was Follow-Up Long Enough for Outcomes to Occur (0 to 1)	Yes (at least one week).	1
		Less than one week or no description.	0
	Adequacy of Follow Up of Cohorts (0 to 1)	At least 80% of subjects complete the follow-up, and subjects lost to follow-up are unlikely to introduce bias.	1
		Follow up rate < 80% and no description of subjects lost to follow-up.	0

Supplementary Material

Table S3. Quality assessment form for cohort studies

Study	Selection				Comparability		Outcome			Total Score
	Representativeness of the Exposed Cohort (0 to 1)	Selection of the Non-Exposed Cohort (0 to 1)	Ascertainment of Exposure (0 to 1)	Demonstration That Outcome of Interest Was Not Present at Start of Study (0 to 1)	Matched or Adjusted by Key Factors (0 to 1)	Matched or Adjusted by Additional Factors (0 to 1)	Assessment of Outcome (0 to 1)	Was Follow-Up Long Enough for Outcomes to Occur (0 to 1)	Adequacy of Follow Up of Cohorts (0 to 1)	
Klok	1	1	1	1	1	0	1	1	1	8
Helms	1	1	1	1	1	1	1	1	1	9
Llitjos	1	1	1	1	1	0	1	0	0	6
Maatman	1	1	1	1	1	1	1	1	1	9
Poissy	1	1	1	1	0	0	1	0	1	6
Artifoni	1	1	1	1	1	1	1	1	1	9
Demelo-Rodríguez	0	1	1	1	1	1	1	1	1	8
Lodigiani	1	1	1	1	1	1	1	1	1	9
Middeldorp	1	1	1	1	1	1	1	1	1	9
Tang	1	1	1	1	0	1	1	0	1	7
Zhang	1	1	1	1	1	1	1	1	1	9

Table S4. Summary of Egger's test for small-study effect

Endpoint	z	P-value
VTE	-0.1343	0.8932
PE	-1.8676	0.0618
DVT	-3.2302	0.0012
Mortality	-1.3295	0.1837

Supplementary Material

Figure S1. PRISMA flow diagram

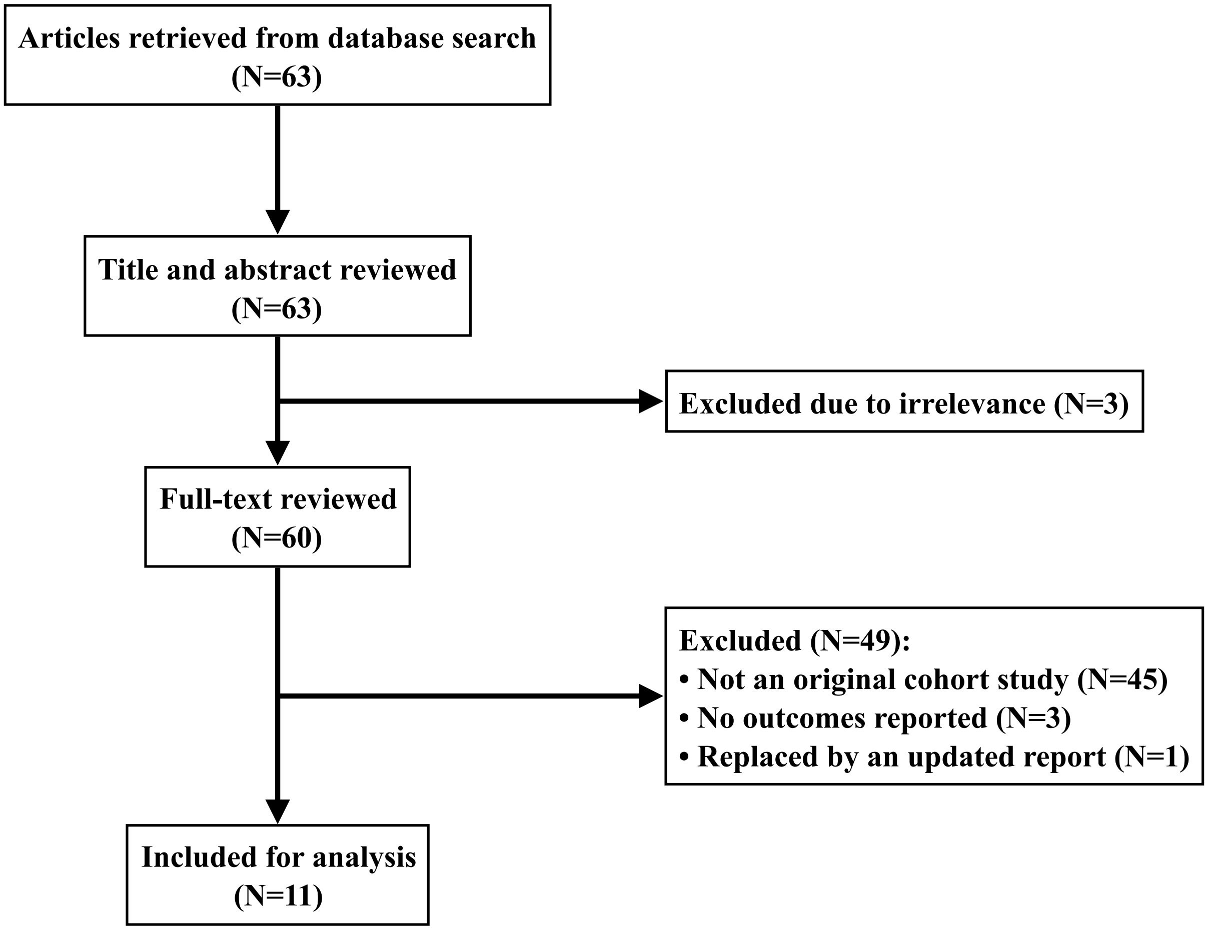

Supplementary Material

Figure S2. Funnel plot of VTE

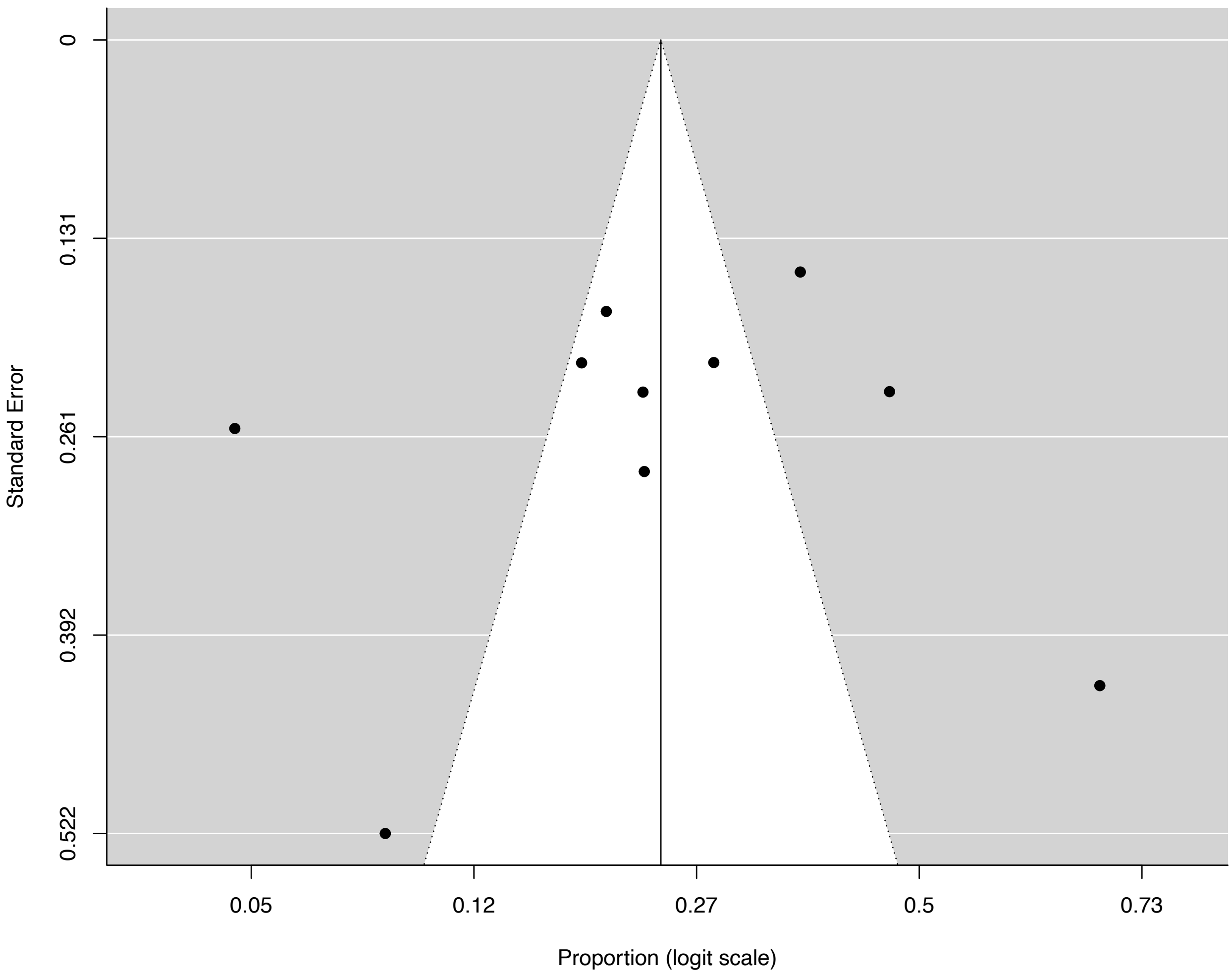

Supplementary Material

Figure S3. Funnel plot of PE

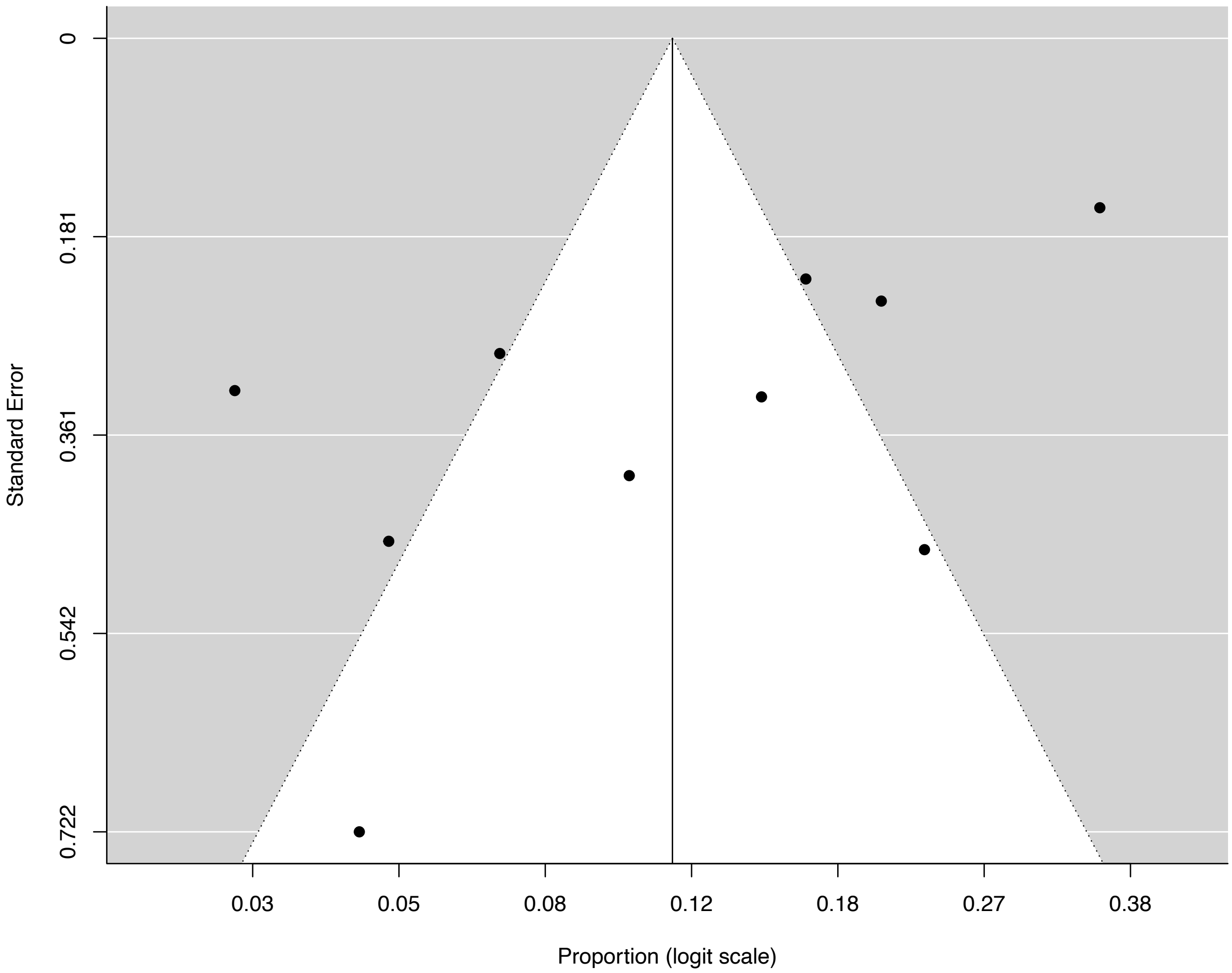

Supplementary Material

Figure S4. Funnel plot of DVT

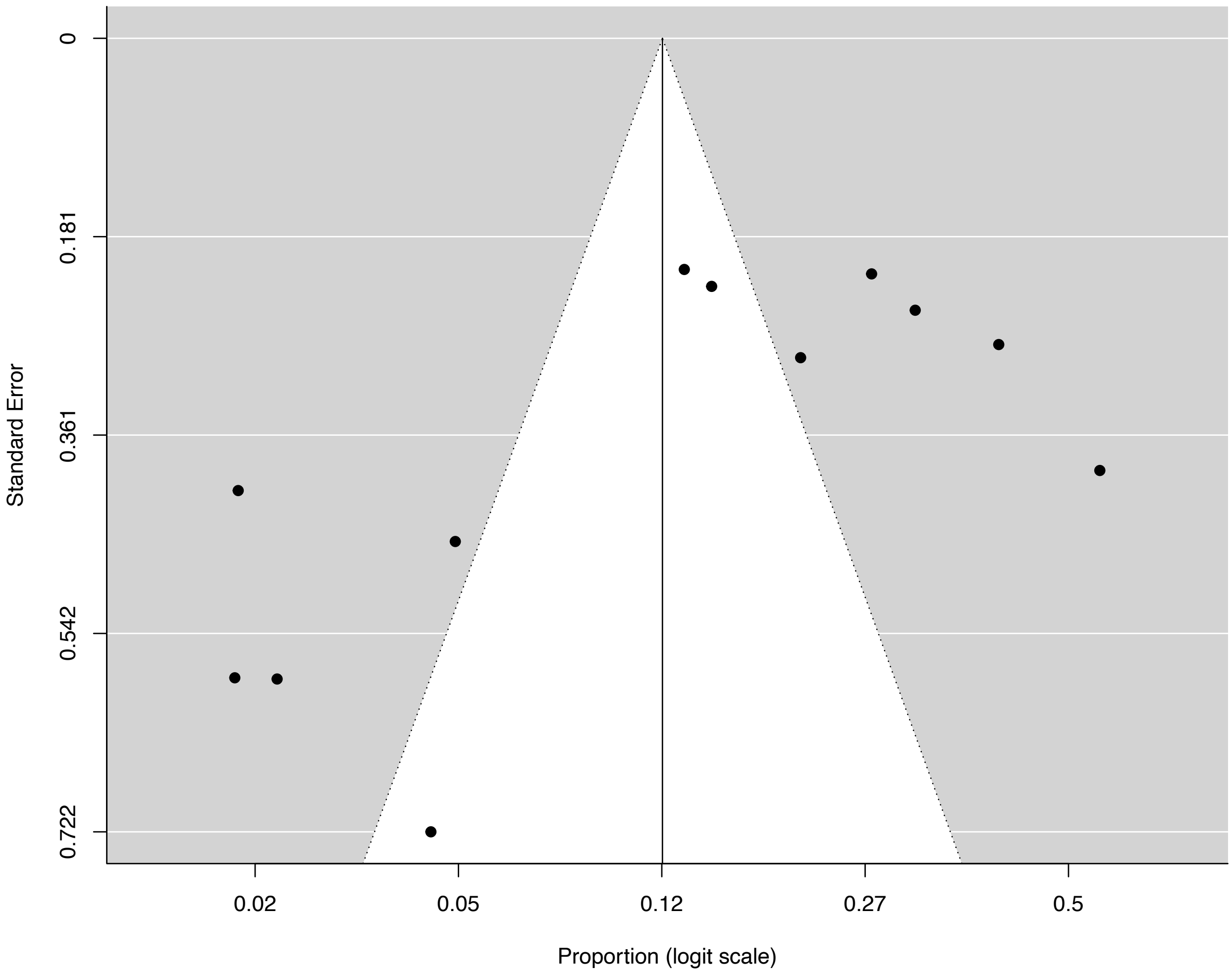

Supplementary Material

Figure S5. Rate of mortality among COVID-19 patients undergoing thromboprophylaxis

Study	Events	Total	Proprotion	95% C.I.
Klok	41	184	0.223	[0.168; 0.289]
Llitjos	3	26	0.115	[0.038; 0.303]
Maatman	27	109	0.248	[0.176; 0.337]
Poissy	15	107	0.140	[0.086; 0.220]
Middeldorp	38	198	0.192	[0.143; 0.253]
Tang	30	99	0.303	[0.221; 0.400]
**Random effects model**		**.**	**0.213**	**[0.170; 0.264]**

Heterogeneity: $I^2 = 53\%$, $\tau^2 = 0.0609$, $\chi^2_5 = 10.75$ ($p = 0.06$)

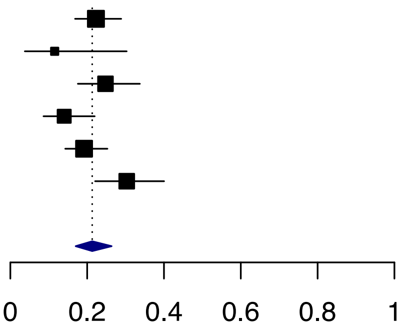

Supplementary Material

Figure S6. Funnel plot of mortality

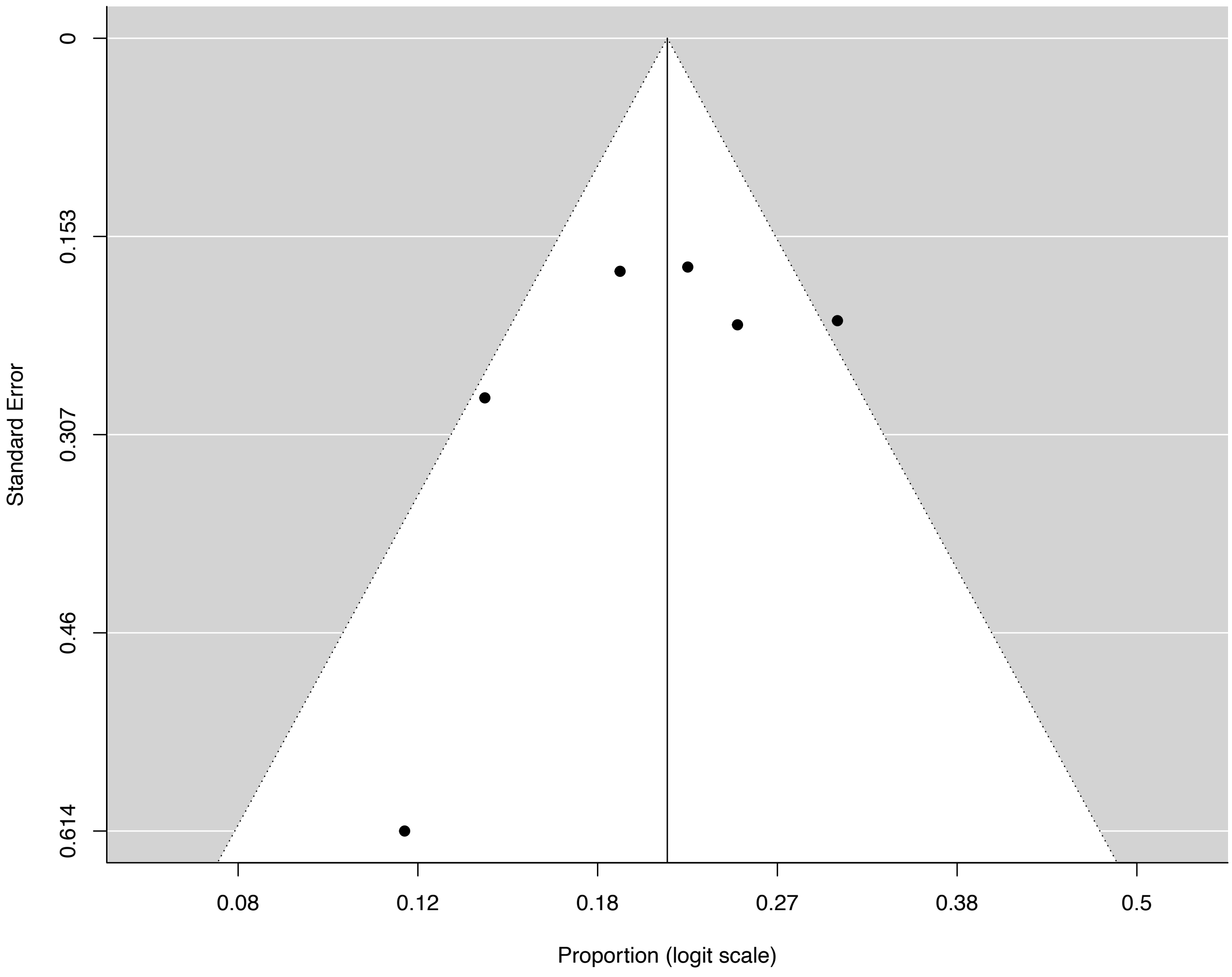

Supplementary Material

Figure S7. Mean difference in D-dimer between cases and controls

	Case			Control				Mean Difference
**Study or Subgroup**	**Mean**	**SD**	**Total**	**Mean**	**SD**	**Total**	**Weight**	**IV, Random, 95% CI**
**1.1.1 VTE**								
Artifoni 2020	2.477	3.316	16	0.747	0.51	55	21.4%	1.73 [0.10, 3.36]
Maatman 2020	1.634	2.467	25	0.5	0.359	59	25.1%	1.13 [0.16, 2.11]
Middeldorp 2020	7.233	13.007	39	1.133	0.748	159	9.6%	6.10 [2.02, 10.18]
**Subtotal (95% CI)**			**80**			**273**	**56.1%**	**2.05 [0.30, 3.80]**
Heterogeneity: $Tau^2 = 1.40$; $Chi^2 = 5.49$, df = 2 (P = 0.06); $I^2 = 64\%$								
Test for overall effect: Z = 2.30 (P = 0.02)								
**1.1.2 Mortality**								
Lodigiani 2020	1.15	1.229	70	0.391	0.26	215	27.6%	0.76 [0.47, 1.05]
Tang 2020	9.04	14.674	134	2.137	2.517	315	16.3%	6.90 [4.40, 9.40]
**Subtotal (95% CI)**			**204**			**530**	**43.9%**	**3.70 [−2.32, 9.72]**
Heterogeneity: $Tau^2 = 18.05$; $Chi^2 = 22.89$, df = 1 (P < 0.00001); $I^2 = 96\%$								
Test for overall effect: Z = 1.21 (P = 0.23)								
**Total (95% CI)**			**284**			**803**	**100.0%**	**2.57 [1.01, 4.14]**
Heterogeneity: $Tau^2 = 2.29$; $Chi^2 = 30.50$, df = 4 (P < 0.00001); $I^2 = 87\%$								
Test for overall effect: Z = 3.22 (P = 0.001)								
Test for subgroup differences: $Chi^2 = 0.27$, df = 1 (P = 0.61), $I^2 = 0\%$								

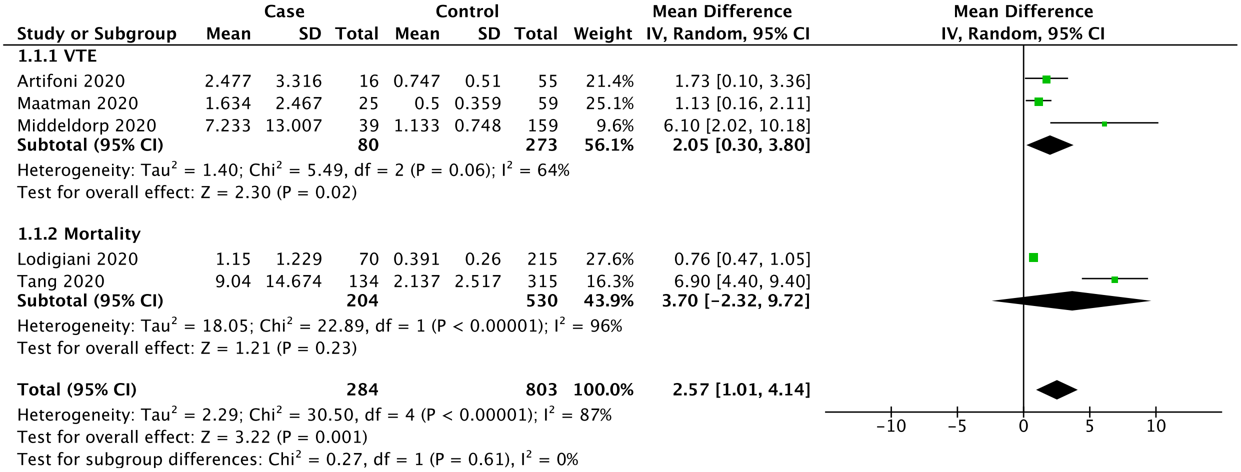

Figure S8. Summary of VTE incidence by clinical setting

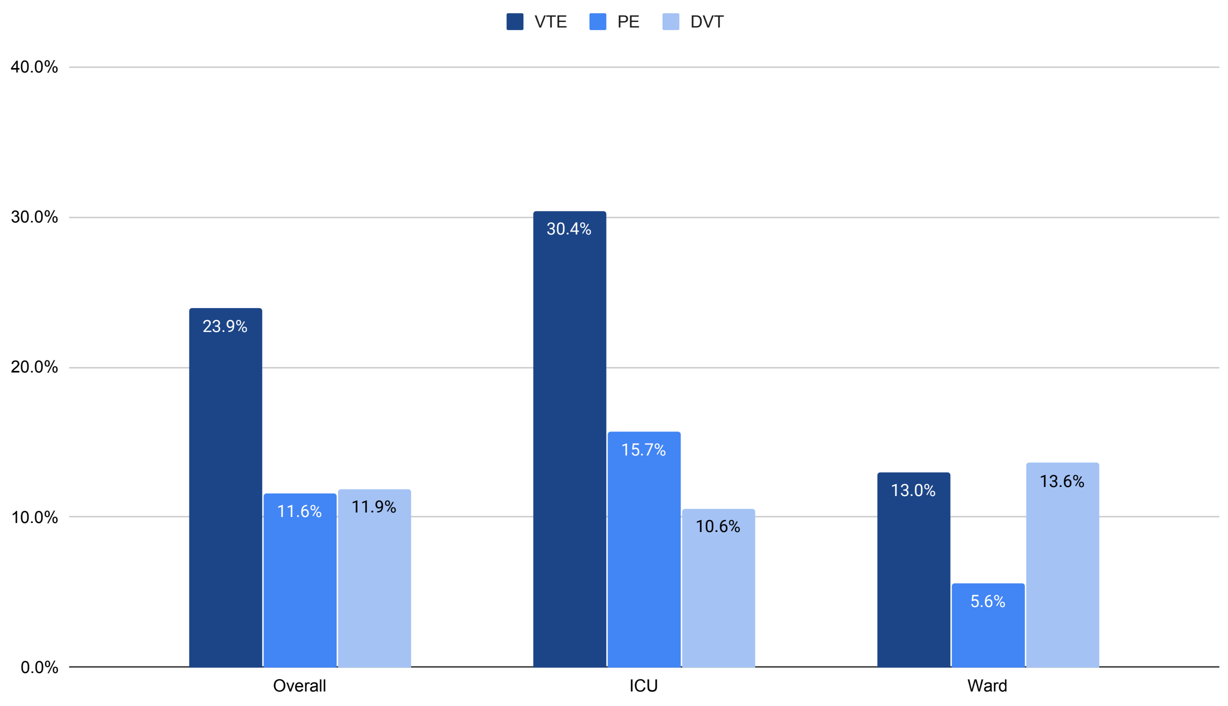

Supplement: Supplementary file 1 [file jcm-09-02489-s001.pdf]
